# Effects of a non‐pharmacological program combining physical and cognitive interventions in older adults in Panama

**DOI:** 10.1002/alz70860_098577

**Published:** 2025-12-23

**Authors:** Elianne S Pauli Quiros, Diana C Oviedo, Gabrielle B Britton, Hjalmar Jones, Jemila Juárez, Alcibiades E Villarreal, Giselle Rangel, Sofía A Rodriguez, Stephanie Lammie, Samantha Miranda, Maria Fernanda Martínez, Emely Ortega, Amy Betancourt, Natalia López

**Affiliations:** ^1^ Instituto de Investigaciones Científicas y Servicios de Alta Tecnología (INDICASAT AIP), Centro de Neurociencias, Panama, Panama; ^2^ Universidad Santa Maria La Antigua, Panama, Panama; ^3^ Instituto de Investigaciones Científicas y Servicios de Alta Tecnología (INDICASAT AIP), Centro de Neurociencias, Panamá, Panamá, Panama; ^4^ Sistema Nacional de Investigación (SNI), Panamá, Panamá, Panama; ^5^ Universidad de Panamá, Panama, Panama; ^6^ Servicio de Neurología, CIDELAS‐CSS, Panamá, Panamá, Panama

## Abstract

**Background:**

Currently, pharmacological interventions to delay cognitive decline in older adults have proven to be insufficient. Non‐pharmacological approaches, however, are gaining attention for improving cognition, enhancing well‐being, and alleviating caregiver burden. This study evaluates the effects of a non‐pharmacological program combining physical and cognitive interventions on cognition, well‐being, and physical health in older adults in Panama.

**Methods:**

This three‐arm randomized controlled trial included 43 participants aged 60‐80. Pre‐tests involved sociodemographic interviews, clinical scales, cognitive tests, and physical assessments. Participants were randomized into three groups: 1) experimental group 1 (*n* = 15) received cognitive and physical interventions, including CogniFit training, group cognitive training, and walks 3 to 5 times weekly; 2) experimental group 2 (*n* = 15) participated exclusively in walks; and 3) active control group (*n* = 13) attended monthly health information sessions. Both experimental groups joined monthly group walks. After the intervention, a post‐test was carried out applying the same initial measures.

**Results:**

Improvements in performance on neuropsychological tests were observed when comparing pre‐ and post‐test scores for the three groups. The combined group showed significant improvements in global cognition and the executive function of abstraction. Improvements were observed also for the combined group in depression and quality of life measures. The physical intervention group presented improvements in learning, visuospatial skills, and long‐term memory. In the control group, increases in learning and the executive function of inhibition were observed. No significant differences were found between pre‐ and post‐tests in physical assessments for any group.

**Conclusion:**

The results obtained suggest that a multidomain intervention program can positively impact cognition, depressive symptoms, and quality of life in older adults. Likewise, physical activity may benefit cognition. This study could serve as a basis for the implementation of multimodal interventions at the community level aimed at the prevention of cognitive decline.

